# Left atrial reservoir strain as a predictor of cardiac outcome in patients with heart failure: the HaFaC cohort study

**DOI:** 10.1186/s12872-022-02545-5

**Published:** 2022-03-14

**Authors:** Sjoerd Bouwmeester, Jonna A. van der Stam, Saskia L. M. van Loon, Natal A. W. van Riel, Arjen-Kars Boer, Lukas R. Dekker, Volkher Scharnhorst, Patrick Houthuizen

**Affiliations:** 1 Department of Cardiology, Catharina Hospital Eindhoven, Michelangelolaan 2, 5623 EJ Eindhoven, The Netherlands; 2 Clinical Laboratory, Catharina Hospital Eindhoven, Eindhoven, The Netherlands; 3 Department of Biomedical Engineering, Computational Biology, Eindhoven University of Technology, Eindhoven, The Netherlands; 4 Expert Center Clinical Chemistry Eindhoven, Eindhoven, The Netherlands; 5 Department of Vascular Medicine, Amsterdam University Medical Centers, Amsterdam, The Netherlands; 6 Department of Electrical Engineering, Eindhoven University of Technology, Eindhoven, The Netherlands

**Keywords:** Left atrial strain, HFrEF, HFmrEF, HFpEF, NT-proBNP

## Abstract

**Background:**

The left atrium (LA) is a key player in the pathophysiology of systolic and diastolic heart failure (HF). Speckle tracking derived LA reservoir strain (LAS_r_) can be used as a prognostic surrogate for elevated left ventricular filling pressure similar to NT-proBNP. The aim of the study is to investigate the correlation between LAS_r_ and NT-proBNP and its prognostic value with regards to the composite endpoint of HF hospitalization and all-cause mortality within 1 year.

**Methods:**

Outpatients, sent to the echocardiography core lab because of HF, were enrolled into this study. Patients underwent a transthoracic echocardiographic examination, commercially available software was used to measure LAS_r_. Blood samples were collected directly after the echocardiographic examination to determine NT-proBNP.

**Results:**

We included 174 HF patients, 43% with reduced, 36% with mildly reduced, and 21% with preserved ejection fraction. The study population showed a strong inverse correlation between LAS_r_ and log-transformed NT-proBNP (r = − 0.75, *p* < 0.01). Compared to NT-proBNP, LAS_r_ predicts the endpoint with a comparable specificity (83% vs. 84%), however with a lower sensitivity (70% vs. 61%).

**Conclusion:**

LAS_r_ is inversely correlated with NT-proBNP and a good echocardiographic predictor for the composite endpoint of hospitalization and all-cause mortality in patients with HF.

*Trial registration***:** https://www.trialregister.nl/trial/7268

## Background

Heart failure (HF) is among the leading causes of morbidity and mortality worldwide [1]. Early recognition and prompt treatment of heart failure are crucial for the prognosis. Although HF is primarily a clinical diagnosis, N-terminal pro-B-type natriuretic peptide (NT-proBNP) is a valuable diagnostic marker of HF given the fact that symptoms can be aspecific [2]. Patients with normal NT-proBNP levels are unlikely to have HF, contrary to patients with elevated levels who need further cardiac evaluation.

Echocardiography is the modality of choice to establish the diagnosis of HF. The classification is based primarily on measurement of left ventricular ejection fraction (LVEF) into HF with reduced (≤ 40%), mildly reduced (41–49%), or preserved (≥ 50%) ejection fraction [3]. Currently, the evaluation of HF is mainly focused on the left ventricle (LV). This is remarkable since left atrial (LA) volume and function has a pathophysiological significance in different types of HF [4, 5]. Accumulating evidence suggests an added value of measuring the left atrial reservoir strain (LAS_r_) by speckle-tracking echocardiography for both diagnosis and prognosis of HF [6, 7]. Previous studies showed that LAS_r_ is around 40% in healthy controls [8] and that it is impaired in HF patients [6, 7]. Additionally, LAS_r_ can be used as a prognostic marker similar to NT-proBNP [9].

The aim of the study is to investigate the correlation between LAS_r_ and NT-proBNP and its prognostic value in an outpatient population with HF with regards to the composite endpoint of HF hospitalization and all-cause mortality within 1 year.

## Methods

### Study design

The present study was performed as part of the Heart Failure Classification (HaFaC) project (https://www.trialregister.nl/trial/7268). This prospective, non-randomized, observational, single-center study was designed to develop a HF classification based on objective measurement data. The local ethics committee and the Institutional Review Board approved the study (Medical Research Ethics Committees United study number NL60579.100.17) and all subjects gave written informed consent. The primary outcome was a composite of all-cause mortality or hospitalization for heart failure.

### Population

From December 2017 to September 2019, patients referred to the Echocardiography Lab with HF based on the ESC guidelines [10] were prospectively included in the study. To be included, patients needed to be ≥ 18 years old and able to provide written informed consent. Exclusion criteria were: recent cardiothoracic surgery (≤ 90 days) or pregnancy. Patients were also excluded for further analysis in case of inadequate acoustic LA window on echocardiography (> 2 non-visible LA segments), and severe renal failure (glomerular filtration rate ≤ 30 mL/min, calculated by the CKD-EPI formula). Also patients with atrial fibrillation were excluded, because atrial fibrillation on its own induces LA remodeling and influences LAS_r_. All patient data was entered into a prospective database, including demographical, clinical and echocardiographic variables, medications and laboratory biomarkers.

### Echocardiographic evaluation

All patients underwent a comprehensive transthoracic echocardiographic examination using commercially available equipment (Philips iE33 or Philips EPIQ, Andover, MA, USA). Examinations were performed by 2 experienced and EACVI certified cardiac sonographers (SB or PH), blinded to other research data. Echocardiogram was stored as Digital Imaging and Communications in Medicine (DICOM) file on a secured server and analysed off-line using commercially available software (QLAB 13, Philips Healthcare, Eindhoven, the Netherlands). Standard 2D- and Doppler-echocardiographic measurements were performed following ASE/EACVI guidelines [11]. LVEF was calculated using the modified biplane Simpson’s rule and maximum LA volume was calculated by the biplane method of disks at end-systole and indexed to body surface area (LAVI). The following parameters were used to determine diastolic dysfunction; average E/e’ > 14, septal e’ velocity < 7 cm/s or lateral e’ velocity < 10 cm/s, tricuspid regurgitation velocity > 2.8 m/s, LAVI > 34 ml/m2, pulmonary vein S/D ratio < 1, mitral inflow velocities and ratio according to the published guidelines [11].

**Fig. 1:**
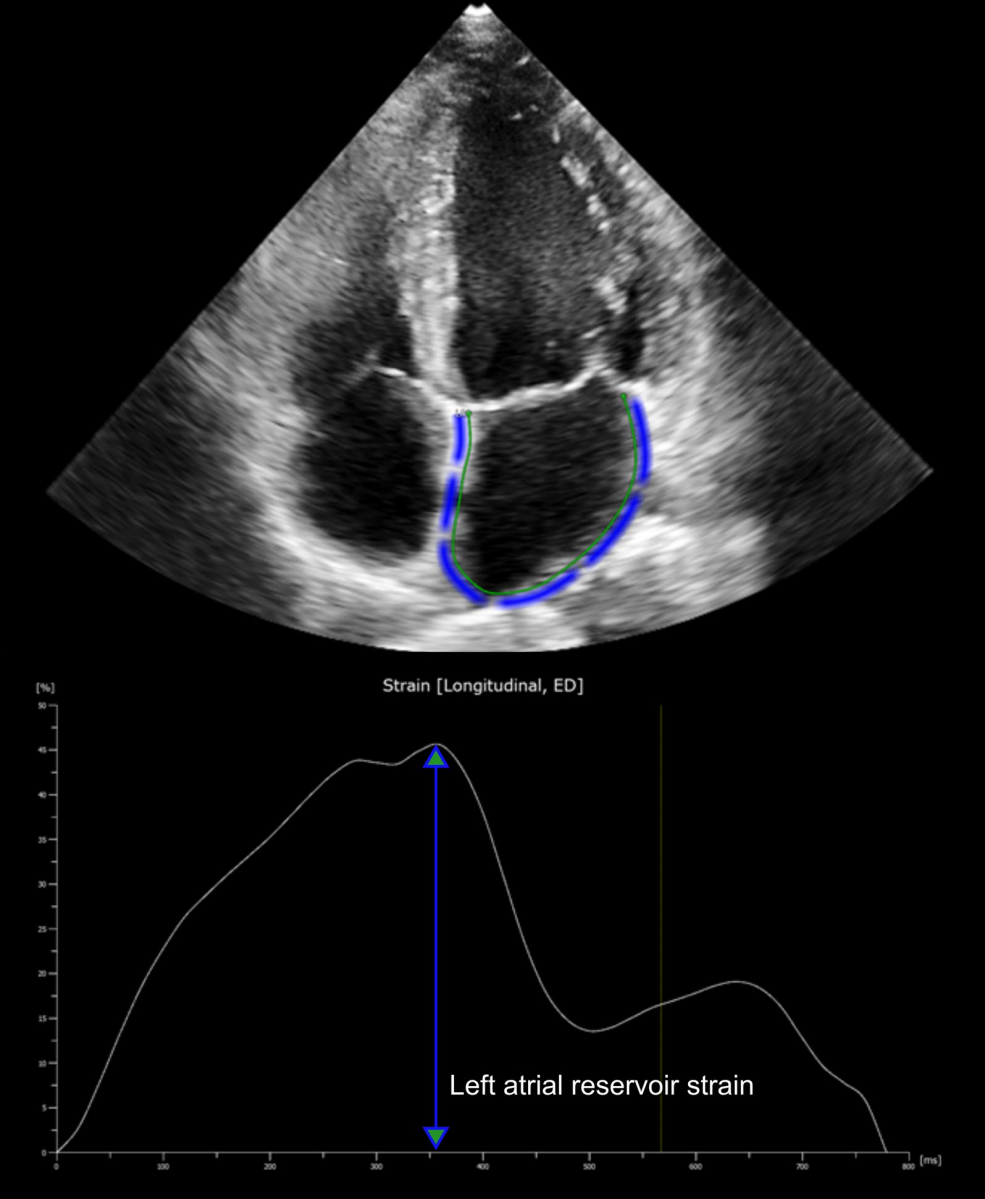
Assessment of left atrial reservoir strain by 2D speckle-tracking echocardiography. This figure shows a non-foreshortened apical four-chamber view of the left atrium. Zero strain reference was set at end-diastole. Arrow represents left atrium reservoir strain (LAS_r_)

Speckle tracking echocardiography of the LA is a relatively new echocardiographic method. With dedicated software an unique pattern of speckles is identified within the LA wall and these speckles are tracked on frame-by-frame base throughout the cardiac cycle. The measured change in distance between the different speckles is used to calculate LA deformation. LA reservoir strain is a prognostic biomarker, which has been evaluated for patients with HF. Commercially available software (QLAB 13, Philips Healthcare, Eindhoven, the Netherlands) was used to measure LAS_r_ on non-foreshortened apical four- and two-chamber views of the LA with a frame rate of 60–80 frames per seconds. The LA endocardial border was automatically drawn followed by manual adjustment if required. The reference point for LA strain analysis was taken at the onset of the QRS-complex (R-R gating) (Fig. 1) [12].

### Biomaker analysis

Blood samples were collected and analyzed directly after the echocardiographic examination while the patient was still in a supine position. Levels of NT-proBNP were determined at the department of Clinical Chemistry (Elecsys pro BNP II assay, Roche Diagnostics, Mannheim, DE) [13].

### Follow-up

Patients were followed up at our outpatient clinic on a regular base by both clinical visits and telephone calls. All-cause mortality was recorded by consulting the Dutch civil registry. Information on HF hospitalization during the 1-year follow-up period was obtained from a systematic review of all hospital admissions performed by an independent reviewer unaware of clinical and echocardiographic data.

### Statistical analysis

Three groups were defined: HF with preserved ejection fraction (HFpEF), HF with mildly reduced ejection fraction (HFmrEF), and HF with reduced ejection fraction (HFrEF) [10]. For continuous variables, normality of distribution was assessed with the Shapiro–Wilk’s test. Normal and skewed continuous variables are presented as means with standard deviation (SD) and medians with interquartile range [IQR], respectively. Statistical comparisons of the three HF subgroups were made using one way ANOVA for normally distributed data or an Kruskal Wallis test for non-normally distributed data. Categorical variables were expressed as proportions and compared using a chi-squared test, or Fishers exact test when the number of positive cases in at least one of the heart failure categories is less than five. Multiple pairwise-comparison between subgroups was performed using Tukey Honest Significant Differences method for normally distributed continuous variables, *p *values of the other multiple comparisons were corrected using Benjamini–Hochberg correction. A *p *value of less than 0.05 was considered to indicate statistical significance. The correlation between LAS_r_ and NT-proBNP were examined by Pearson’s correlation analysis. Prognostic value of the different parameters was assessed by a receiver-operator curve (ROC-curve), the optimal cut-off point was determined by maximizing the Youden Index. Kaplan–Meier curves are shown for the time-to-event distribution. All analyses were performed using R version 4.0.5 and Rstudio 1.2.1335 (R foundation for Statistical Computing, Vienna, Austria; RStudio InC, Boston, MA).

## Results

### Patient selection

**Fig. 2:**
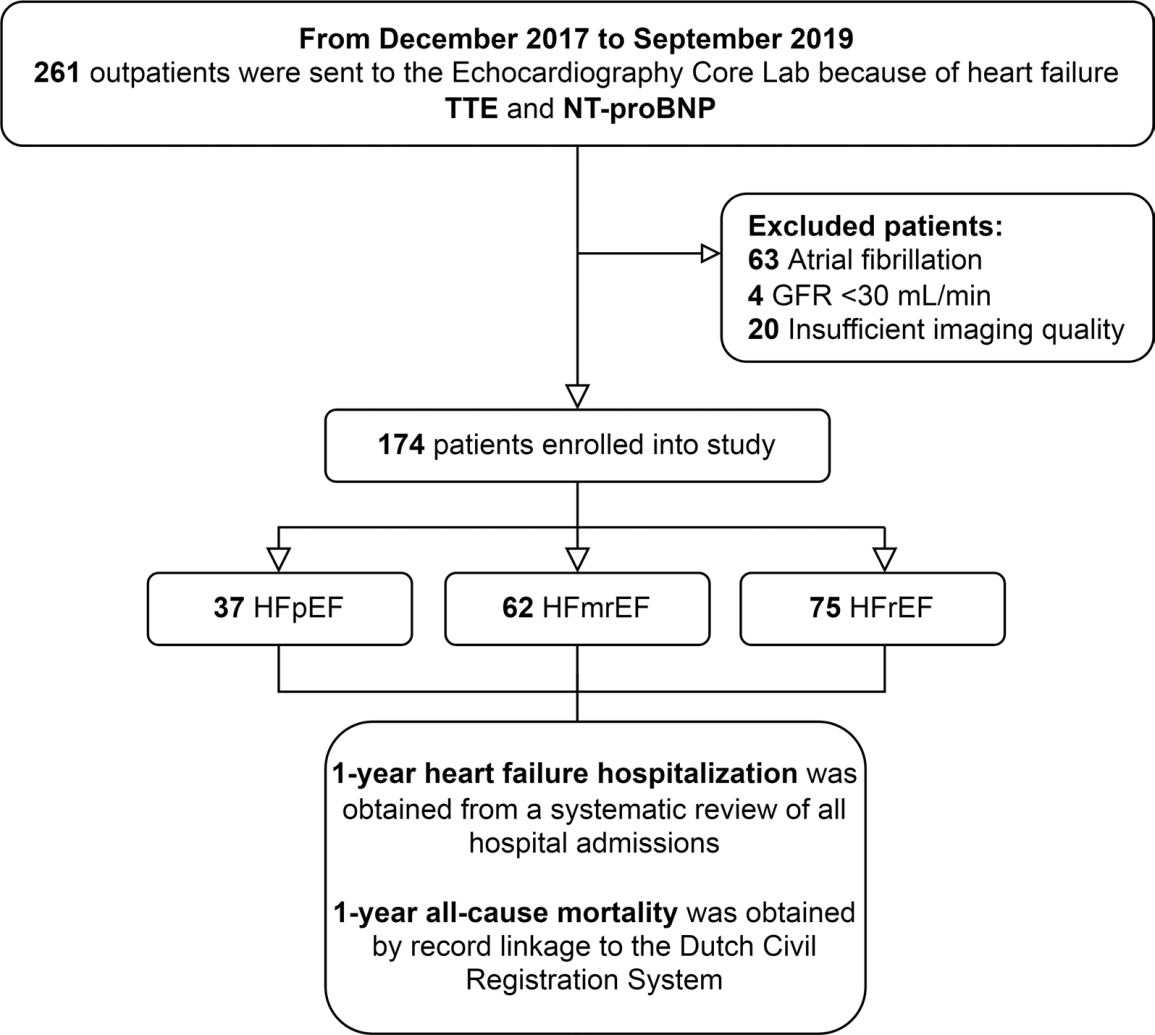
Flow-chart of patient selection. HFrEF, heart failure with reduced ejection fraction; HFmrEF, heart failure with mildly reduced ejection fraction; HFpEF, heart failure with preserved ejection fraction; GFR, glomerular filtration rate

Two hundred sixty-one outpatients were sent to the echocardiography Core Lab because of HF. Eighty-seven patients were excluded because of atrial fibrillation (n = 63), severe renal failure (n = 4) and insufficient imaging quality for LAS_r_ analysis (n = 20). The remaining 174 patients were enrolled into the study; there were 37 patients with HFpEF (21%), 62 with HFmrEF (36%), and 75 with HFrEF (43%) (Fig. 2).

### Baseline characteristics

Table 1 shows general characteristics of the total study population and HF subgroups. Patients were predominantly male (69%) with a median age of 68 years. The majority of patients were treated with beta blockers (79%), renin-angiotensin system antagonists (79%), and to a lesser extent with mineralocorticoid antagonists (31%) and loop diuretics (41%).

**Table 1:** General characteristics

	Total group (n = 174)	HFpEF (n = 37)	HFmrEF (n = 62)	HFrEF (n = 75)	*p *value
*Baseline demographics*					
Age—yr	68 [59–75]	74 [70–80]	62 [56–68]	68 [60–76]	** < 0.01**
Male	120 [69%]	18 [49%]	42 [68%]	60 [80%]	** < 0.01**
Body mass index—kg/m2	26 [24–29]	28 [26–31]	26 [24–28]	26 [24–29]	**0.03**
Hypertension	88 [51%]	28 [76%]	28 [45%]	32 [43%]	** < 0.01**
Systolic blood pressure—mmHg	132 [118–145]	142 [132–164]	131 [120–141]	129 [116–141]	** < 0.01**
Diastolic blood pressure—mmHg	76 [71–85]	79 [70–88]	79 [72–86]	74 [71–80]	0.13
Heart Rate—bpm	66 [58–78]	68 [60–82]	69 [59–75]	65 [57–80]	0.76
Diabetes Mellitus	26 [15%]	3 [8%]	8 [13%]	15 [20%]	0.23
COPD	21 [12%]	8 [22%]	6 [10%]	7 [9%]	0.14
Chronic kidney disease	21 [12%]	6 [16%]	4 [6%]	11 [15%]	0.23
Coronary artery disease	67 [39%]	6 [16%]	23 [37%]	38 [51%]	** < 0.01**
*NYHA functional class*					
I	61 [37%]	8 [24%]	33 [54%]	20 [28%]	** < 0.01**
II	62 [37%]	13 [38%]	17 [28%]	32 [44%]	0.14
III	40 [24%]	11 [32%]	9 [15%]	20 [28%]	0.09
IV	4 [2%]	2 [6%]	2 [3%]	0 [0%]	0.08
*Heart failure medication*					
Beta-blocker	137 [79%]	26 [70%]	50 [81%]	61 [82%]	0.31
Renin-angiotensin system antagonist	137 [79%]	26 [70%]	52 [84%]	59 [80%]	0.27
Mineralocorticoid antagonist	54 [31%]	6 [16%]	19 [31%]	29 [39%]	0.05
Neprilysine Inhibitor	5 [3%]	0 [0%]	2 [3%]	3 [4%]	0.72
Loop diuretics	71 [41%]	17 [46%]	16 [26%]	38 [51%]	**0.01**
*Endpoints*					
Heart Failure Hospitalization	18 [10%]	3 [8%]	3 [5%]	12 [16%]	0.09
Mortality	11 [6%]	3 [8%]	1 [2%]	7 [9%]	0.12
Composite endpoint	23 [13%]	4 [11%]	3 [5%]	16 [21%]	**0.02**

*p* values < 0.05 are shown as bold to indicate statistical significance

On echocardiography, median LVEF was 44% [34–49], median left ventricular end-diastolic volume (LVEDV) 142 ml [100–195], and median LAVI 37 ml/m^2^ [28–47] with a median LAS_r_ 27% [20–35]. On subgroup analysis, patients with HFrEF had significantly larger LVEDV and prevalence of mitral valve regurgitation was higher compared with HFmrEF (*p* < 0.01). LA size did not differ significantly between HFrEF and HFpEF patients, however LAS_r_ was lower in the HFrEF compared with HFpEF and HFmrEF patients (*p* = 0.02 and *p* < 0.01, respectively) (Table 2).

**Table 2:** Echocardiographic and laboratory parameters

	Total group (n = 174)	HFpEF (n = 37)	HFmrEF (n = 62)	HFrEF (n = 75)	*p *value
*LV parameters*					
LVEDV—ml	142 [100–195]	92 [68–115]	134 [98–168]	194 [151–230]	**< 0.01**
LVESV—ml	82 [52–121]	38 [30–48]	70 [53–94]	128 [102–164]	**< 0.01**
LV mass index—gram/m2	106 [89–132]	94 [82–120]	101 [86–121]	120 [97–139]	** < 0.01**
LVEF—%	44 [34–49]	57 [56–60]	47 [45–48]	33 [26–38]	** < 0.01**
LA parameters					
LAVI—ml/m2	37 [28–47]	39 [32–46]	32 [25–40]	41 [34–52]	**< 0.01**
LAS_r_—%	27 [20–35]	27 [24–32]	33 [26–39]	23 [14–30]	**< 0.01**
RV parameters					
TAPSE—cm	2 [0.47]	2.1 [0.48]	2.0 [0.40]	1.9 [0.50]	0.08
Peak TR gradient—mmHg*	26 [20–34]	30 [26–38]	21 [18–5]	29 [22–36]	** < 0.01**
*Valvular heart disease*					
Aortic stenosis ≥ moderate	6 [3%]	2 [5%]	0 [0%]	4 [5%]	0.12
Aortic regurgitation ≥ moderate	3 [2%]	0 [0%]	2 [3%]	1 [1%]	0.60
Mitral stenosis ≥ moderate	0 [0%]	0 [0%]	0 [0%]	0 [0%]	-
Mitral regurgitation ≥ moderate	31 [18%]	5 [14%]	4 [6%]	22 [29%]	<** 0.01**
Tricuspid regurgitation ≥ moderate	8 [5%]	1 [3%]	0 [0%]	7 [9%]	**0.02**
*Laboratory biomarkers*					
CKD-EPI—mL/min	68 (22)	59 (16)	74 (21)	67 (23)	**< 0.01**
NT-proBNP—pg/mL	568 [276–1114]	568 [298–925]	357 [138–706]	788 [449–1913]	**< 0.01**

*p* values < 0.05 are shown as bold to indicate statistical significance

LVEDV = left ventricular end-systolic volume, LVESV = left ventricular end-systolic volume, LVEF = left ventricular ejection fraction, LAVI = left atrial volume index, LAS_r_ = left atrial reservoir strain, TAPSE = tricuspid annular plane systolic excursion, TR = tricuspid regurgitation, CKD-EPI = Chronic Kidney Disease Epidemiology Collaboration, NT-proBNP = N-terminal pro-B-type natriuretic peptide, *data available in 75 patients (39%)

Median NT-proBNP (568 pg/mL [276–1114]) was significantly higher in the HFrEF group (HFmrEF *p* < 0.01, HFpEF *p* = 0.02) (Table 2).

### *Correlation between biomarkers and LAS*_*r*_

**Fig. 3:**
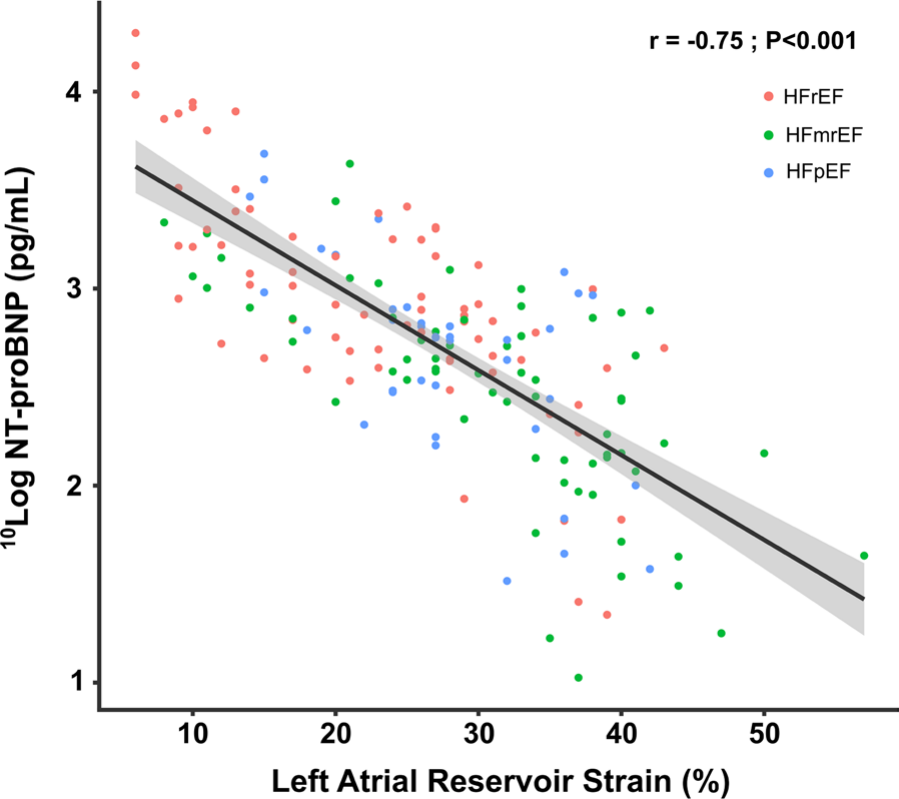
Correlation between NT-proBNP and LAS_r_. Scatter plots showing an inverse correlation of NT-proBNP and left atrial reservoir strain in the overall group. r, Pearsons correlation coefficient

The study population showed a moderate inverse correlation between NT-proBNP and LAS_r_ (r = − 0.55 *p* < 0.01), which improved after ^10^log-transformation of NT-proBNP (r = − 0.75, *p* < 0.01) (Fig. 3). For the HF subgroups, no significant differences were found between the degree of correlation between ^10^log-transformed NT-proBNP and LAS_r_.

### *Correlation between conventional echocardiographic diastolic parameters and LAS*_*r*_

Both deceleration time of early mitral inflow (E) and early diastolic mitral annular velocity (e’) had a weak correlation with LAS_r_ (r = 0.35 and r = 0.24, respectively). E/A and E/e’ ratio had a moderate inverse correlation with LAS_r_ (r = − 0.44 and r = − 0.42, respectively).

### Follow-up

Twenty-three patients (13%) reached the composite endpoint of all-cause mortality and heart failure hospitalization (Table 1).

### *Prognostic value of LAS*_*r*_* and biomarkers*

**Fig. 4:**
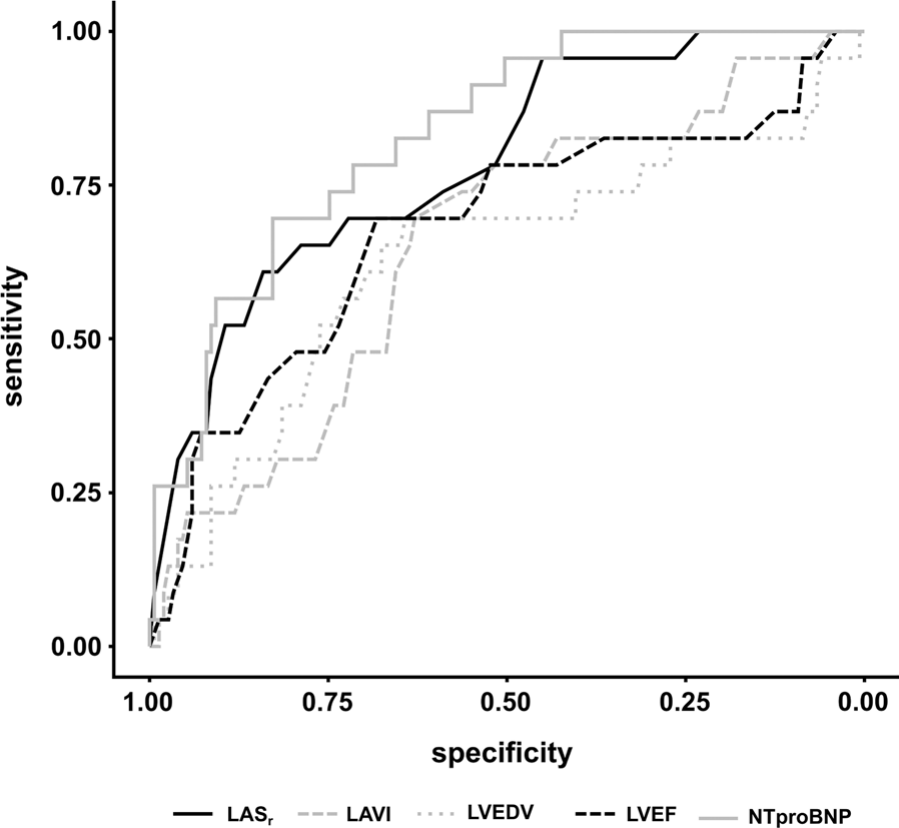
Receiver operating characteristic analysis. Receiver operating characteristic analysis for each parameter against the primary endpoint (HF hospitalization and all-cause mortality)

**Fig. 5:**
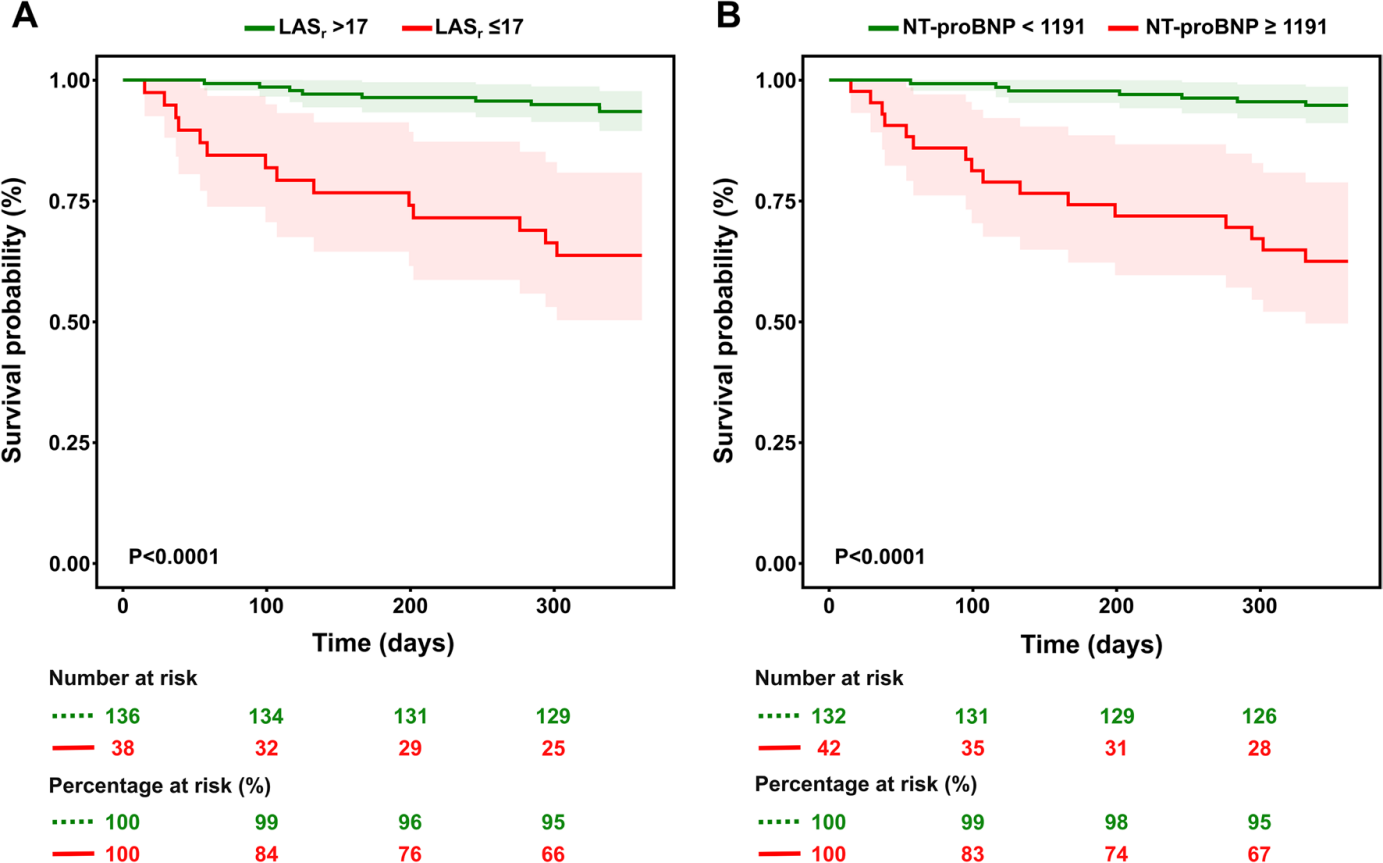
Kaplan–Meier survival curve. Kaplan–Meier survival curves according to LAS_r_ with cut-off ≤ 17% (Panel **A**) and NT-proBNP with cut-off < 1191 pg/mL (Panel **B**). Composite endpoint: HF hospitalization and all-cause mortality

Results of receiver operating characteristic (ROC) analysis for all predictors of the endpoint are shown in Fig. 4 and Table 3. NT-proBNP showed the highest area under the ROC curve (AUC 0.83) to predict the primary endpoint of death or heart failure hospitalization up to 12 months of follow-up. As for echocardiographic parameters, LAS_r_ outperformed LVEF with a AUC-value of 0.79. The AUC of LAS_r_ differed significantly from LAVI and LVEDV (*p* < 0.01 and *p* = 0.03), however not from LVEF and NT-proBNP (*p* = 0.10 and *p* = 0.25). Figure 5 shows survival curves by Kaplan Meier analysis for patients stratified by LAS_r_ (Panel A) and NT-proBNP (Panel B). Patients with LAS_r_ ≤ 17% showed significantly worse survival than patients with LAS_r_ > 17%. Patients with NT-proBNP ≥ 1191 pg/mL also showed significantly worse survival.

**Table 3:** Receiver operating characteristic to predict adverse events

	Cut-off	AUC	Sensitivity (%)	Specificity (%)
*Echocardiographic parameters*				
LVEDV—ml	≥ 166	0.63	70	64
LVEF—%	≤ 38	0.68	70	68
LAVI—ml/m2	≥ 40	0.65	70	63
LAS_r_—%	≤ 17	0.79	61	84
*Laboratory parameter*				
NT-proBNP—pg/mL	≥ 1191	0.83	70	83

LVEDV = left ventricular end-systolic volume, LVESV = left ventricular end-systolic volume, LVEF = left ventricular ejection fraction, LAVI = left atrial volume index, LAS_r_ = left atrial reservoir strain, NT-proBNP = N-terminal pro-B-type natriuretic peptide

## Discussion

The key findings of the present study are as follows. First, LAS_r_ is a strong echocardiographic predictor of the composite endpoint of HF hospitalization and all-cause mortality. Compared to NT-proBNP, LAS_r_ predicts the endpoint with a comparable specificity (83% vs. 84%), however with a lower sensitivity (70% vs. 61%). Also, LAS_r_ correlates strongly with ^10^log-transformed NT-proBNP levels.

LAS_r_ has enhanced prognostic value beyond conventional echocardiographic measures to discriminate which heart failure patients are at greater risk for hospital admission or death. For HF subgroups, Carluccio et al. [15] and Freed et al. [16] showed that assessment of LAS_r_ by speckle-tracking strain echocardiography had powerful prognostication in patients with HFrEF and HFpEF, respectively.

Although limited in number, previous studies that investigated the correlation between NT-proBNP and LAS_r_, are in line with our results. Al Saikhan [7] demonstrated a modest inverse correlation in patients with both HFpEF (r = − 0.57) and HFmrEF (r = − 0.53). Another study showed that LAS_r_ had moderate inverse correlation with NT-proBNP (r = − 0.42) [14]. In both studies, the correlation between LAS_r_ and a log-transformation of NT-proBNP levels was not reported, although it has been shown that plasma concentrations of NT-proBNP follow a log-normal distribution in patients with HF [13]. Prastaro [5] evaluated the relationship between NT-proBNP and LA function in patient with HFrEF. In their study, LA function was based on measuring fractional active and total emptying from M- and B-mode images and showed a significant correlation between NT-proBNP and LA function [5].

### LA reservoir strain and elevated LV filling pressure

Assessment of LV filling pressure has important diagnostic and prognostic implications in patients with HF [10, 11]. Although right-sided cardiac catheterization is the gold standard to determine LV filling pressure, it is unattractive for routine clinical use given its invasiveness. In the continuing search for non-invasive markers to estimate LV filling pressure, NT-proBNP provides a reliable estimation, especially for left ventricular end-diastolic pressure (LVEDP) [17]. The prognostic value of NT-proBNP is well established [9, 10] and the results of our study are in line with previous reports. The LA, on the other hand, is more and more acknowledged as key player in the pathophysiology of systolic and diastolic HF [18]. Indeed, elevated LV filling pressure results in pressure overload that induces LA failure characterized by dilation and decrease in reservoir function. Wakami [19] showed that an increase in LVEDP is associated with a decrease in LAS_r_. Moreover, LAS_r_ can accurately categorize patients based on a normal or elevated LV filling pressures [14, 20].

### LA reservoir strain and heart failure

In HFpEF a fortiori, elevated filling pressures are the main physiologic consequence of the diastolic dysfunction [21]. Current guidelines [11] use various echocardiographic parameters for determination of diastolic dysfunction. LAS_r_ provides potentially clinical relevance in the detection of LV diastolic dysfunction, because LAS_r_ detects subtle dysfunction, even before the LA begins to enlarge [22]. LAS_r_ decreases in a linear fashion as LV diastolic dysfunction progresses [23]. In line with this, the latest EACVI document [24] encourages the use of LA strain in the assessment of diastolic function and filling pressures in HFpEF, however LAS_r_ should not be used in patients with atrial fibrillation [25]. We previously showed a relationship between increased coronary microvascular resistance and reduced LAS_r_, that seemed to precede conventional measures of LV diastolic dysfunction [26]. Moreover, LAS_r_ is not only influenced by diastolic, but also by systolic LV function. As LA expansion is also determined by the base-to-apex displacement during LV systolic contraction [27], any condition that influences LV myocardial function is expected to influence LAS_r_. Thus, LAS_r_ correlates with both LV filling pressures and systolic performance.

### Clinical implications

The LA seems to have a central role in HF. LA function can be easily studied using speckle-tracking strain echocardiography. The LA strain measurements should be included in the standard evaluation of outpatients with heart failure, because it can stratify their risk for hospital admission and death more reliable than LVEF. Further research is need if a closer follow-up of these patients will reduce their morbidity and mortality.

### Study limitations

Although the current study is based on a real-world, prospective, observational data of an outpatient HF population referred to the echocardiography lab, the subgroups of different HF types were relatively small. Second, the number of events was too small to perform a multivariate Cox regression analysis. A logistic regression with NT-proBNP and echocardiographic parameters did not result in an improvement in AUC. However, follow-up research with a larger population and more events is needed to draw conclusions on the AUC of combined laboratory and echocardiographic parameters. Third, the length of follow-up in the current study was limited to 1 year, so we were unable to determine the long-term prognostic value of LAS_r_. Fourth, in this prospective study a combined endpoint of HF hospitalization and all-cause mortality has been chosen. Cardiovascular death might have been a stronger endpoint. However, the exact cause of death was not known in all of our HF patients. Finally, in 7% of the study population, LAS_r_ could not be analyzed due to poor image quality.

## Conclusions

LAS_r_ is a strong echocardiographic predictor of the composite endpoint of HF hospitalization and mortality. LAS_r_ is inversely correlated with NT-proBNP and predicts the endpoint with a comparable specificity.

## Data Availability

The datasets used and/or analysed during the current study are available from the corresponding author on reasonable request.

## Abbreviations

HF: Heart failure
HFmrEf: Heart failure with mildly reduced ejection fraction
HFpEF: Heart failure with preserved ejection fraction
HFrEF: Heart failure with reduced ejection fraction
LA: Left atrial
LAS_r_: Left atrial reservoir strain
LAVI: Left atrial volume indexed to body surface are
LV: Left ventricle
LVEDP: Left ventricular end-diastolic pressure
LVEDV: Left ventricular end-diastolic volume
LVEF: Left ventricular ejection fraction
NT-proBNP: N-terminal pro-B-type natriuretic peptide
